# Interferon-γ induces interleukin-6 production by neutrophils via the Janus kinase (JAK)-signal transducer and activator of transcription (STAT) pathway

**DOI:** 10.1186/s13104-021-05860-w

**Published:** 2021-12-11

**Authors:** Shuhei Yoshida, Shunya Yamada, Kohei Yokose, Haruki Matsumoto, Yuya Fujita, Tomoyuki Asano, Naoki Matsuoka, Jumpei Temmoku, Shuzo Sato, Makiko Yoshiro-Furuya, Hiroshi Watanabe, Kiyoshi Migita

**Affiliations:** Department of Rheumatology, Fukushima Medical University School of Medicine, 1 Hikarigaoka, Fukushima, Fukushima 960-1295 Japan

**Keywords:** Cytokines, Interferon-γ, Interleukin-6, Janus kinase (JAK), Neutrophils, Rheumatoid arthritis

## Abstract

**Objective:**

Interferon-gamma (IFN-γ) is overexpressed in rheumatoid synovium and thought to be involved in the pathogenesis of rheumatoid arthritis (RA). In this study, we examined our hypothesis that IFN-γ activates innate immune cells and upregulates inflammatory cytokines. Peripheral blood neutrophils were stimulated with IFN-γ in the presence or absence of Janus kinase (JAK) inhibitors. Interleukin-6 (IL-6) mRNA and protein expression were analyzed using real-time polymerase chain reaction (PCR) method and enzyme-linked immunosorbent assay. Protein phosphorylation of JAKs or STAT1 was assessed by Western blot using phospho-specific antibodies.

**Results:**

IFN-γ stimulation induces IL-6 expression in protein and mRNA levels in human neutrophils. Furthermore, IFN-γ stimulation induces JAK1/JAK2 phosphorylation and downstream signal transducer and activator of transcription (STAT) 1 phosphorylation in human neutrophils. Although all JAKi, blocked IFN-γ-induced JAK1.2/STAT1 phosphorylation at higher concentrations (100 nM), baricitinib most efficiently inhibited IFN-γ-induced JAK1.2/STAT1 phosphorylation at lower concentrations (≤ 25 nM). Among these JAKi, baricitinib was the most potent regulator for IFN-γ-induced IL-6 production in human neutrophils. Our data indicate that IFN-γ upregulates IL-6 production via the JAK1/2-STAT1 pathway in human innate immune cells. Furthermore, this IFN-γ-mediated IL-6 induction via JAK/STAT was downregulated by JAKi.

**Supplementary Information:**

The online version contains supplementary material available at 10.1186/s13104-021-05860-w.

## Introduction

Interferon-gamma (IFN-γ) is involved in the regulation of both the innate and adaptive immune responses [1]. The major biological activities of IFN-γ include activation of cellular immunity [2]. IFN-γ activates innate immune cells and induces major histocompatibility complex (MHC) class II antigen expression, possibly contributing to the development of rheumatoid arthritis (RA) [3]. A clinical trial using an anti-IFN-γ monoclonal antibody for RA was terminated since it did not meet the primary endpoint [4]. In contrast, Janus kinase (JAK) inhibitors targeting multiple cytokines, including IFN-γ, exhibit a beneficial effect in RA [5]. In RA synovium, CD8^+^ T cells are thought to be a source of IFN-γ, which leads to downstream cytokine cascades through by activating rheumatoid synovial infiltrating cells [6, 7].

An important role of IFN-γ is the regulation of immune cell growth and differentiation by activating the transcription of target genes [8]. IFN-γ signaling pathway is mediated by activating Janus kinase (JAK) and signal transducer and activator of transcription (STAT) 1 [9]. After binding to its receptor, IFN-γ activates the receptor-associated JAK1 and JAK2 by phosphorylation [10]. The activated JAKs allow for subsequent phosphorylation of STAT1, which then dissociates from the IFN-γ receptor, translocates to the nucleus and binds to the promoter regions of IFN-γ-inducible genes [11]. JAK inhibitors (JAKi) target multiple cytokines, including interleukin-6 (IL-6) or granulocyte–macrophage colony-stimulating factor (GM-CSF), and exhibit clinical efficacy in the treatment of RA [12]. However, the molecular mechanisms for the negative regulation of IFN-γ-induced signal transduction pathways by JAKi have not been completely elucidated. The aim of the present study was to investigate whether IFN-γ activates innate immune cells to produce proinflammatory cytokines.

## Materials and methods

### Reagents

Recombinant human IFN-γ was purchased from Peprotech (Rocky Hills, NJ). Phospho-specific antibodies against JAK-1 (Tyr1022/1023), JAK-2 (Tyr1007/1008) and STAT-1 (Tyr705) were purchased from Cell Signaling Technology (Beverly, MA, USA). Tofacitinib, baricinib and upadacitinib were purchased from Sigma-Aldrich (Tokyo, Japan).

### Neutrophils isolation

Venous peripheral blood was collected from healthy volunteers. Neutrophils were isolated by density gradient centrifugation using Polymorphprep TM (Axis-Shield, Oslo, Norway). Flow cytometric immunophenotyping was performed to assess the purity of the isolated neutrophils. The isolated neutrophils were positively stained with CD11b (96.5%, Additional file 1). The isolated neutrophils (2 × 10^6^/ml) were seeded in 24-well plates containing RPMI1640 with 10% FBS and stimulated with the indicated concentrations of IFN-γ. To investigate the effects of JAKi on IFN-γ receptor signaling, freshly isolated neutrophils were pretreated with JAKi for 1 h then stimulated with IFN-γ and protein extracts or supernatants were analyzed by Western blotting or ELISA. This study (No. 29282) was approved by the Ethics Committee of Fukushima Medical University and written informed consent was obtained from each individual.

### ELISA

Cell-free supernatants were analyzed for IL-6 was measured using enzyme-linked immnunosorbent assay kit (R&D Systems, No. HS600C Minneapolis, MN, USA).

### Reverse transcription-polymerase chain reaction (RT-PCR)

Total RNA was extracted using the RNeasy total RNA isolation protocol (Qiagen, Crauley, UK) according to the manufacturer’s protocol. First-strand cDNA was synthesized from 1 μg of total cellular RNA using an RNA PCR kit (Takara Bio Inc., Otsu, Japan). cDNA was amplified using specific primers respectively.

Primer sets for human IL-6 and GAPDH were purchased from OriGene (Rockville MD, USA). The specific sequences for IL-6 were as follows [13]:

GAPDH: forward primer 5′-GTCTCCTCTGACTTCAACAGCG-3′,

reverse primer 5′-ACCACCCTGTTGCTGTAGCCAA-3′;

IL-6; forward primer 5′-AGACAGCCACTCACCTCTTCAG-3′;

reverse primer 5′-TTCTGCCAGTGCCTCTTTGCTG-3′.

PCR program: Stage 1: Activation: 50 °C for 2 min; Stage 2: pre-soak:95 °C for 10 min; Stage 3: Denaturation: 95 °C for 15 s, Annealing: 60 °C for 1 min; Stage 4: Melting curve 95 °C for 15 s, 60 °C for 15 s, 95 °C for 15 s. PCR was carried out using Real-time PCR Detection System (Applied Biosystems) with relative expression analysis determined by reference to the housekeeping gene GAPDH. The relative expression of the target genes was calculated using the ΔΔCt method.

### Cell lysis and western blotting

Cells were stimulated with IFN-γ for the indicated times in the figure legends and the cells were washed by ice-cold PBS and lysed with RIPA Buffer (Sigma-Aldrich) with 1.0 mM sodium orthovanadate for 20 min at 4 °C. After 5 min on ice, the cell lysates were centrifuged at 10,000*g* for 10 min at 4 °C. After centrifugation, an identical amount of cellular lysates (50 μg) was subjected to electrophoresis, transferred to a nitrocellulose membrane and proved with phospho-specific antibodies (JAK1, JAK2, STAT1). Targeted protein bands were detected using the enhanced chemiluminescence (ECL) system (Amersham, Little Chalfont, UK). Densitometry was done using the automated digitizing software (Image J, NIH, Bethesda, USA). All phosphorylated bands of JAK1, JAK2 and STAT1 were normalized to total protein levels.

### Statistical analysis

Differences between groups were examined for statistical significance using Mann–Whitney U test.

## Results

### IFN-γ induces IL-6 secretion by human neutrophils

We initially examined whether IFN-γ stimulates inflammatory cytokine production by neutrophils. The culture supernatants from IFN-γ-stimulated neutrophils were analyzed for IL-6**,** which is involved in RA pathogenesis, using enzyme-linked immunosorbent assays. IFN-γ stimulated the production of IL-6 from neutrophils in a dose-dependent manner (Additional file 2). We next assessed IL-6 mRNA expression in IFN-γ-stimulated neutrophils. IFN-γ was a potent inducer for IL-6 mRNA expression in neutrophils (Additional file 3). A concentration-dependent increase in the IL-6 mRNA expression was found after stimulation with IFN-γ and reached a plateau at 50 ng/ml of IFN-γ.

### Inhibitory effect of JAKi on IL-6 production

IFN-γ transduces signals via Janus kinase (JAK)/signal transduction activator of transcription (STAT) pathways. In order to investigate the involvements of JAK in IFN-γ-induced IL-6 production, we pretreated freshly isolated neutrophils with various concentrations of JAKi (tofacitinib, baricitinib, upadacitinib) for 1 h and then stimulated IFN-γ for 24 h. We assessed the production of IL-6 from IFN-γ-stimulated neutrophils.

**Fig. 1:**
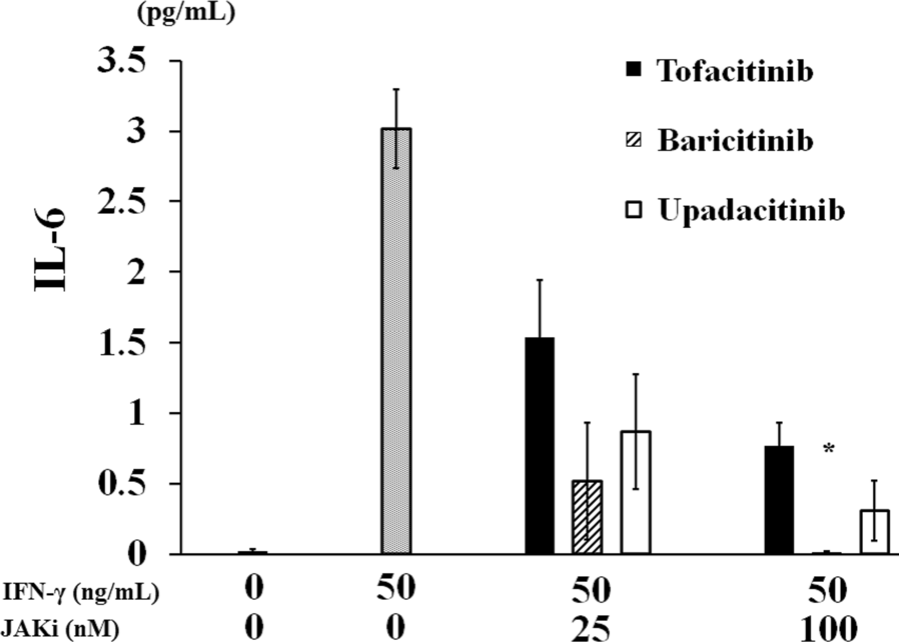
JAKi inhibit the IFN-γ-induced IL-6 synthesis from neutrophils. Neutrophils were pretreated with JAKi (tofacitinib, baricitinib, upadacitinib) at the indicated concentrations for 1 h and then stimulated with IFN-γ (50 ng/ml) for 24 h. Supernatants were analyzed for IL-6 production by ELISA. Values represent the mean ± SD of three independent experiments. *p = 0.015 baricitinib-pretreated neutrophils versus those with tofacitinib. *p* = 0.024 baricitinib-pretreated neutrophils versus those with upadacitinib. Results were compared by the Mann–Whitney U test

As expected IFN-γ-stimulated neutrophils produced a significant amounts of IL-6. Pretreatment of neutrophils with JAKi showed the decrease in IL-6 production in a dose-dependent manner. Whereas, pretreatment of neutrophils with lower concentrations of baricitinib showed the strongest decrease in IL-6 production compared to those with tofacitinib or upadacitinib from IFN-γ-stimulated neutrophils (Fig. 1).

### Inhibitory effect of JAKis on cytokine-induced STAT phosphorylation

**Fig. 2:**
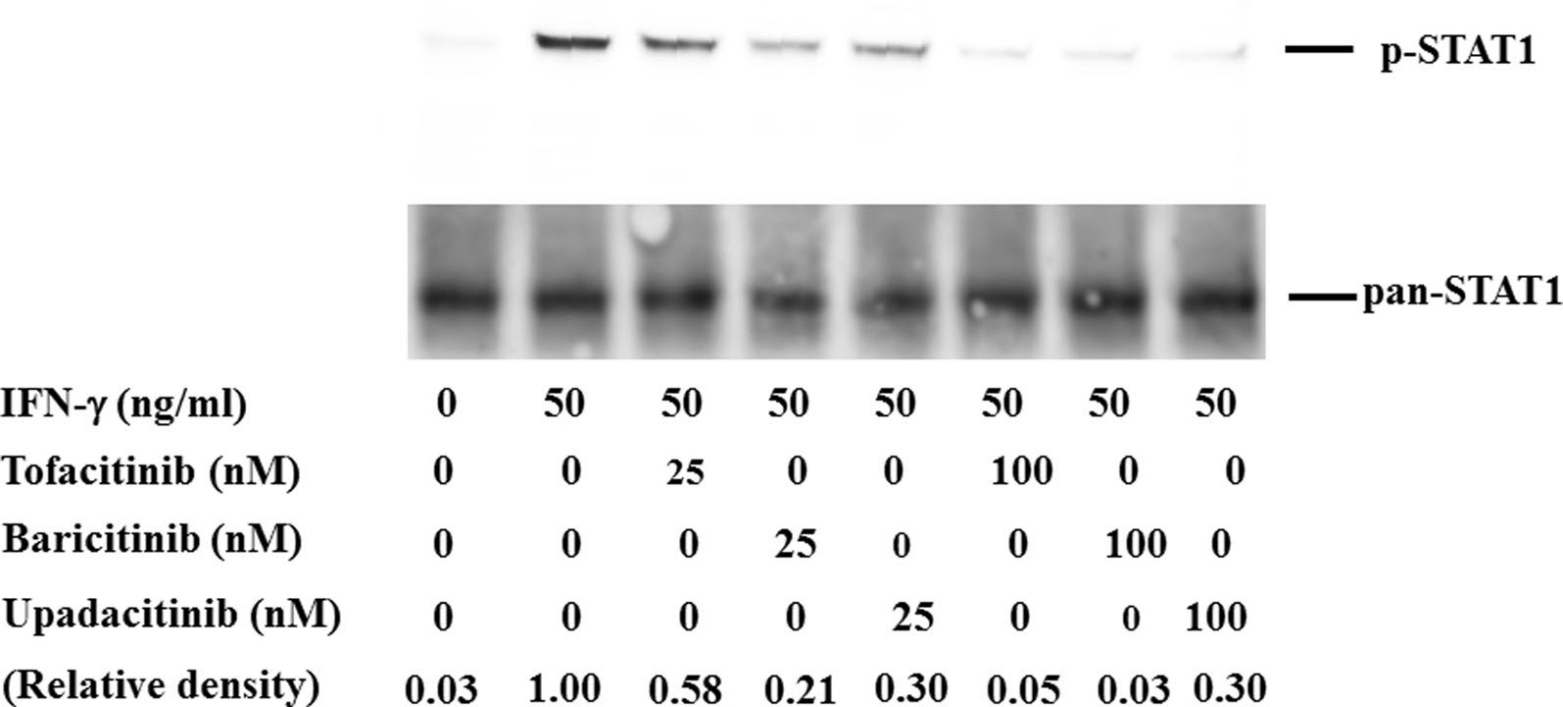
Effects of JAKi on STAT1 phosphorylation in IFN-γ stimulated neutrophils. Neutrophils were pretreated with JAKi (tofacitinib, baricitinib, upadacitinib) at the indicated concentrations for 1 h and then stimulated with IFN-γ (50 ng/ml) for 20 min. Phosphorylation of STAT1 was determined by Western blotting using phospho-specific antibodies against STAT1. Phosphorylation levels of JAK2 were normalized to total protein levels. For relative quantification, the ratio of phosphorylated STAT1 to total STAT1 was defined as 1.0 in the IFN-γ-stimulated neutrophils

**Fig. 3:**
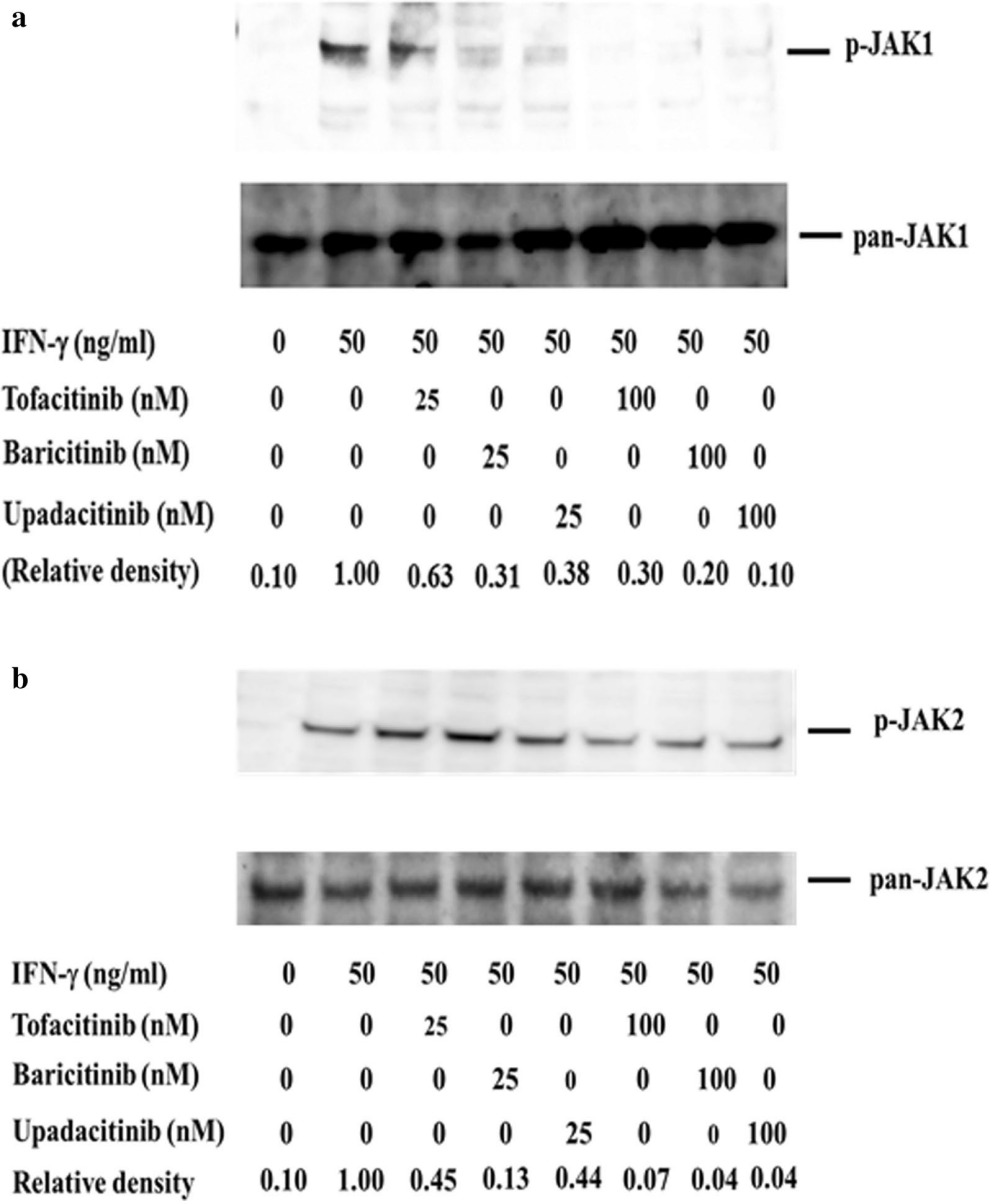
Effects of JAKi on JAK1/2 phosphorylation in IFN-γ stimulated neutrophils. Neutrophils were pretreated with JAKi (tofacitinib, baricitinib, upadacitinib) at the indicated concentrations for 1 h and then stimulated with IFN-γ (50 ng/ml) for 20 min. Phosphorylation of JAK1 (**A**) and JAK2 (**B**) were determined by Western blotting using phospho-specific antibodies against JAK1 and JAK2. Phosphorylation levels of JAK1 or JAK2 were normalized to total protein levels. For relative quantification, the ratio of phosphorylated JAK to total JAK was defined as 1.0 in the IFN-γ-stimulated neutrophils

To compare the inhibitory activity of each JAKi against JAK/STAT signaling pathway, we evaluated IFN-γ-induced STAT1 phosphorylation. Treatment of IFN-γ with 50 ng/ml resulted in the phosphorylation of STAT1 in human neutrophils. Dose-dependent response experiments showed that all JAKis effectively suppressed STAT1 phosphorylation at higher concentrations (100 nM) in IFN-γ-stimulated human neutrophils. While the inhibitory effects of JAKis varied at lower concentrations (25 nM), and baricitinib most efficiently inhibited IFN-γ-induced STAT1 phosphorylation (Fig. 2, Additional file 4). Pretreatment with all JAKi blocked IFN-γ-induced JAK1 phosphorylation at lower or higher concentrations (25–100 nM, Fig. 3, Additional file 5). Although pretreatment with all JAKi blocked IFN-γ-induced JAK2 phosphorylation at higher concentrations (100 nM), The inhibitory effects of JAKi varied at lower concentrations (25 nM). Similarly, baricitinib most efficiently inhibited IFN-γ-induced JAK2 phosphorylation even at lower concentrations (Fig. 3, Additional file 6).

## Discussion

RA is an inflammatory arthritis leading to the joint destruction [14]. Although the etiology of RA is not completely understood, it is known that the cross-talk between adaptive immunity and innate immunity is involved in RA immunopathology [15]. IFN-γ plays an important role in the development of autoimmunity [2]. The high levels of IFN-γ are found in RA plasma, synovial tissue, and synovial fluids [16]. However, INF-γ plays conflicting roles in the development and progression of RA, with some studies highlighting a protective role of IFN-γ in RA [17]. This is because the functions of IFN-γ are dependent on the types of immune cells [18].

This study was conducted to assess the effect of IFN-γ on innate immune cells. We also examined whether JAKi affect the INF-γ-mediated signal transduction pathway. The results demonstrated that IFN-γ stimulation induced the production of an inflammatory cytokines, IL-6, which plays an important role in the cytokine cascade of RA [19]. The mechanism by which IFN-γ upregulates IL-6 expression in neutrophils was not elucidated in this study. It was reported that IFN-γ itself does not stimulate IL-6 production, however, IFN-γ enhances the production of IL-6 in LPS-stimulated THP-I cells [20]. The promoter region of the interleukin-6 (IL-6) gene has a putative NF-kappa B (NF-κB)-binding site [21]. IFN-γ was shown to activate downstream signaling molecules, including NF-κB, in addition to JAK/STAT pathways [22]. It is possible that IFN-γ stimulats human neutrophils to produce IL-6 in which NF-κB is involved in IL-6 gene expressions.

We also examined the effect of JAKi on IFN-γ-induced STAT1 activation, which is involved in the transcription of IFN-γ-induced genes. All JAKis (tofacitinib, baricitinib, and upadacitinib) efficiently suppressed IFN-γ-induced STAT1 phosphorylation. However, the inhibitory effect of tofacitinib seems to be lower compared to those of baricitinib or upadacitinib against IFN-γ-induced STAT1 phosphorylation under the lower concentrations. Our data also indicated that baricitinib is the most potent inhibitor for IFN-γ-induced IL-6 production at lower concentrations (25 nM).

It is assumed that each JAKi exhibits different inhibitory profiles against cytokine receptor signaling according to their specificities for JAK isoforms [23, 24]. Our data indicated that there are differences in the inhibitory effects on the JAK/STAT pathway among different JAKi. Concerning the IFN-γ-induced STATI phosphorylation, tofacitinib showed the lower inhibitory activities compared to baricitinib or upadactinib. This observation contrasts with the comparable inhibition on IFN-γ-induced JAK1 phosphorylation in neutrophils pretreated with these there JAKi. Conversely tofacitinib showed lower inhibitory activities against IFN-γ-induced JAK2 phosphorylation in parallel to the inhibitory effects on IFN-γ-induced STAT1 phosphorylation. These findings may suggest that IFN-γ-induced STAT1 activation process may be more depend on JAK2. Among these three JAKi used in this study, baricitinib is the most strong inhibitor for IFN-γ-mediated signaling pathways in innate immune cells.

Previous basic studies demonstrated that IFN-γ is involved in RA pathogenesis, including synovial inflammation and the activation of innate immunity [25]. However, clinical trials targeting IFN-γ are unsuccessful in the treatment of RA [4], suggesting that biological activities IFN-γ may be variable and depend of the types of immune cells [17]. In rheumatoid synovium, it is presumed that IFN-γ, which is secreted from synovial infiltrating T cells, activates synovial fibroblasts, and monocytes/macrophages [26]. These activated synovial inflammatory cells may produce IL-6, which contribute to rheumatoid synovitis [27]. Our results clearly indicated that IFN-γ activates innate immune cells to produce IL-6 and JAKi regulate this IFN-γ-triggered IL-6 production probably by affecting the STAT1 activation processes.

In conclusion, IFN-γ activates human neutrophils, resulting in STAT1 activation and IL-6 production. Our data also indicated that the IFN-γ-mediated JAK/STAT signaling pathway can be modulated by JAKi resulting in the inhibition of production of IL-6. Inhibition of IFN-γ-mediated inflammatory pathways using JAKis likely plays a role in the modification of rheumatoid inflammatory processes.

### Limitation

There are some variations in IFN-γ-induced IL-6 production in neutrophils. Although we used the freshly isolated neutrophils, the cells have not always exactly the same property due to the variability attributable to individual differences, which may account for these variations of IFN-γ-induced IL-6 productions.

## Supplementary Information

**Additional file 1: Isolated neutrophils are a pure populations.** Isolated neutrophils were collected and stained for CD11b/CD3 and analyzed by flow cytometry. Isolated neutrophils demonstrated a single spot in their light scattering properties (FSC versus SS density plot). Percentages of cells positive for CD3 and CD11b are indicated.

**Additional file 2: IFN-γ induces IL-6 synthesis from human neutrophils.** Neutrophils were incubated with the indicated concentrations of IFN-γ for 24 h and supernatants were analyzed for p IL-6 and TNF-α production by ELISA. Values represent the mean ± SD of three independent experiments.

**Additional file 3: IFN-γ induces IL-6 mRNA expressions in human neutrophils.** Neutrophils were incubated with the indicated concentrations of IFN-γ for 6 h. The cells were harvested and analyzed for IL-6 mRNA levels by real-time PCR. Values represent the mean ± SD of three independent experiments.

**Additional file 4:** **p-STAT1 full blot.** Neutrophils were pretreated with JAKi (tofacitinib, baricitinib, upadacitinib) at the indicated concentrations for 1 h and then stimulated with IFN-γ (50 ng/ml) for 20 min. Phosphorylation of STAT1 was determined by Western blotting using phospho-specific antibodies against STAT1.

**Additional file 5: p-JAK1 full blot.** Neutrophils were pretreated with JAKi (tofacitinib, baricitinib, upadacitinib) at the indicated concentrations for 1 h and then stimulated with IFN-γ (50 ng/ml) for 20 min. Phosphorylation of JAK1 was determined by Western blotting using phospho-specific antibodies against JAK1.

**Additional file 6: p-JAK2 full blot.** Neutrophils were pretreated with JAKi (tofacitinib, baricitinib, upadacitinib) at the indicated concentrations for 1 h and then stimulated with IFN-γ (50 ng/ml) for 20 min. Phosphorylation of JAK2 was determined by Western blotting using phospho-specific antibodies against JAK2.

## Data Availability

The datasets used and analysed during the current study are available from the corresponding author on reasonable request.

## Abbreviations

IFN-γ: Interferon-gamma (IFN-γ)
IL-6: Interleukin-6
JAK: Janus kinase
JAKi: JAK inhibitors
RA: Rheumatoid arthritis (RA)
STAT: Signal transducer and activator of transcription
