# Supplementary figures and images for: Interferon-γ induces interleukin-6 production by neutrophils via the Janus kinase (JAK)-signal transducer and activator of transcription (STAT) pathway
Source: BMC Res Notes. 2021 Dec 11;14:447. doi: 10.1186/s13104-021-05860-w (PMC8666078; doi:10.1186/s13104-021-05860-w)

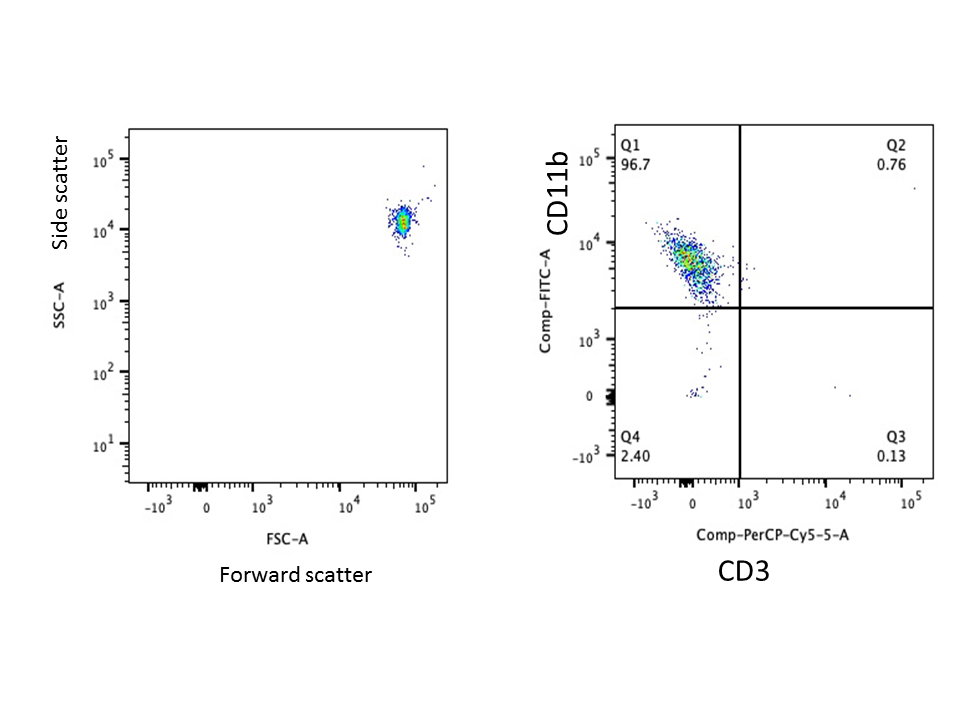

Supplement: Supplementary file 1 — Additional file 1: Isolated neutrophils are a pure populations. Isolated neutrophils were collected and stained for CD11b/CD3 and analyzed by flow cytometry. Isolated neutrophils demonstrated a single spot in their light scattering properties (FSC versus SS density plot). Percentages of cells positive for CD3 and CD11b are indicated. [file 13104_2021_5860_MOESM1_ESM.tif]

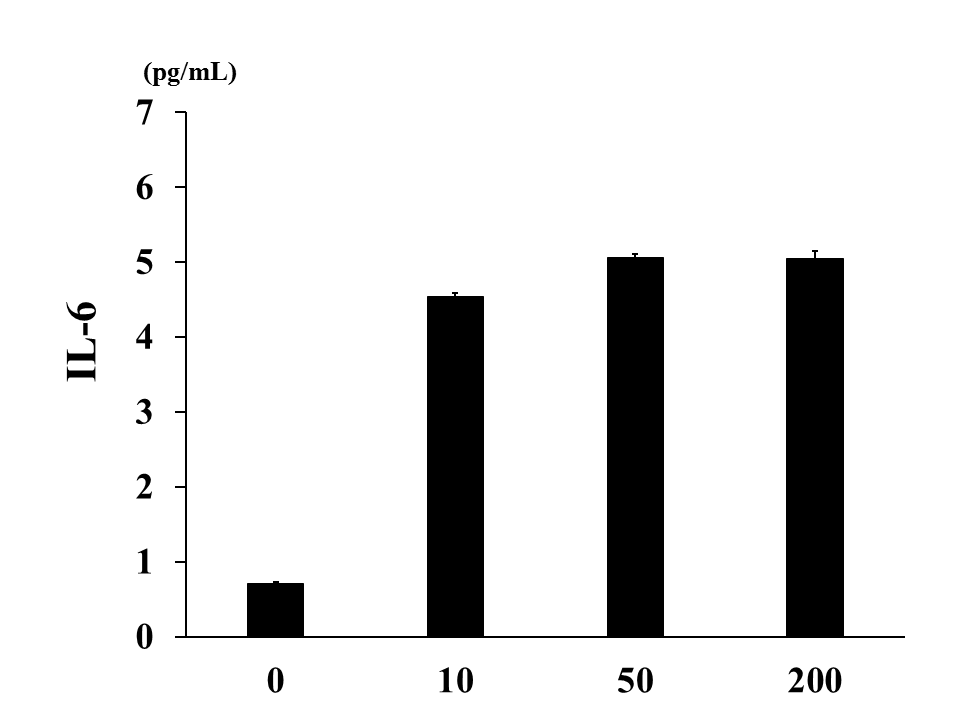

Supplement: Supplementary file 2 — Additional file 2: IFN-γ induces IL-6 synthesis from human neutrophils. Neutrophils were incubated with the indicated concentrations of IFN-γ for 24 h and supernatants were analyzed for p IL-6 and TNF-α production by ELISA. Values represent the mean ± SD of three independent experiments. [file 13104_2021_5860_MOESM2_ESM.tif]

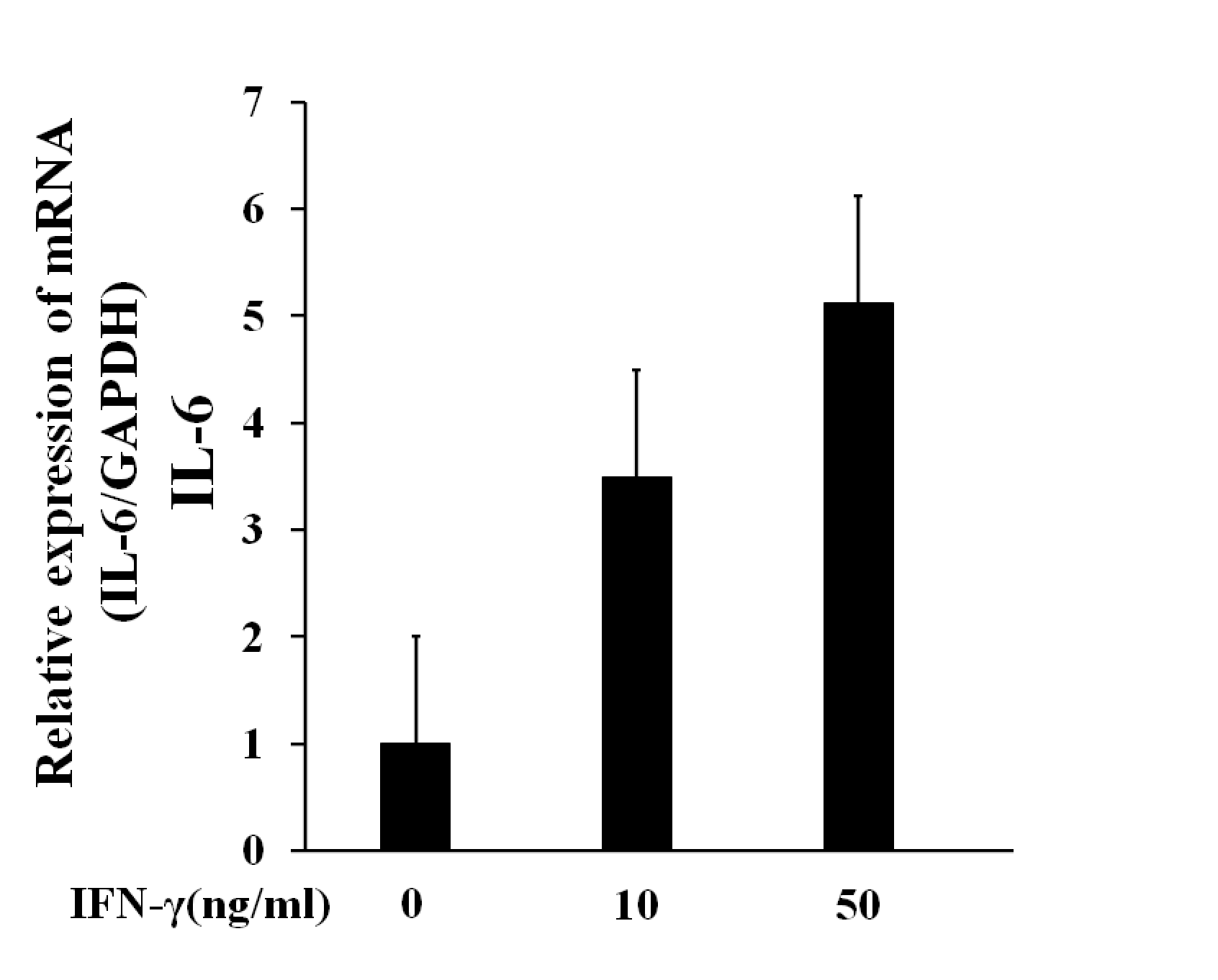

Supplement: Supplementary file 3 — Additional file 3: IFN-γ induces IL-6 mRNA expressions in human neutrophils. Neutrophils were incubated with the indicated concentrations of IFN-γ for 6 h. The cells were harvested and analyzed for IL-6 mRNA levels by real-time PCR. Values represent the mean ± SD of three independent experiments. [file 13104_2021_5860_MOESM3_ESM.tif]

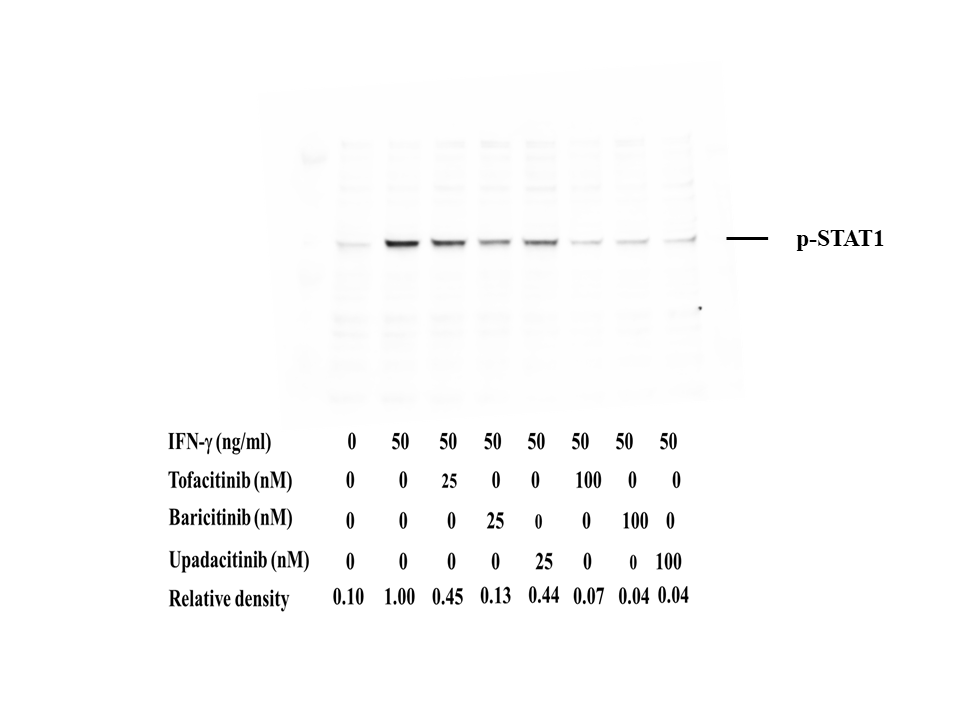

Supplement: Supplementary file 4 — Additional file 4: p-STAT1 full blot. Neutrophils were pretreated with JAKi (tofacitinib, baricitinib, upadacitinib) at the indicated concentrations for 1 h and then stimulated with IFN-γ (50 ng/ml) for 20 min. Phosphorylation of STAT1 was determined by Western blotting using phospho-specific antibodies against STAT1. [file 13104_2021_5860_MOESM4_ESM.tif]

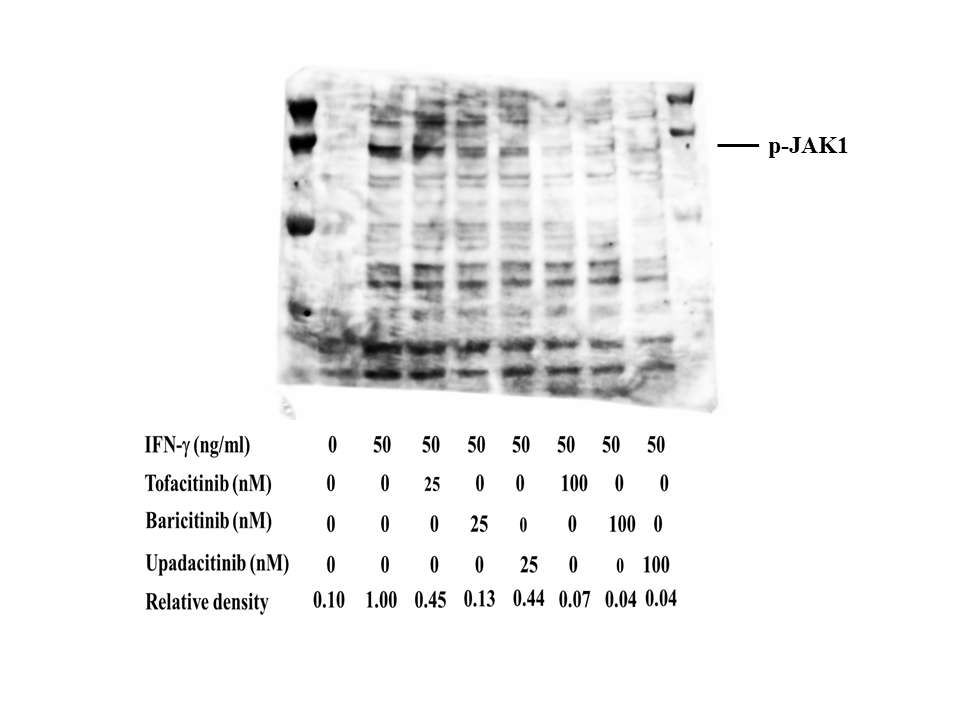

Supplement: Supplementary file 5 — Additional file 5: p-JAK1 full blot. Neutrophils were pretreated with JAKi (tofacitinib, baricitinib, upadacitinib) at the indicated concentrations for 1 h and then stimulated with IFN-γ (50 ng/ml) for 20 min. Phosphorylation of JAK1 was determined by Western blotting using phospho-specific antibodies against JAK1. [file 13104_2021_5860_MOESM5_ESM.tif]

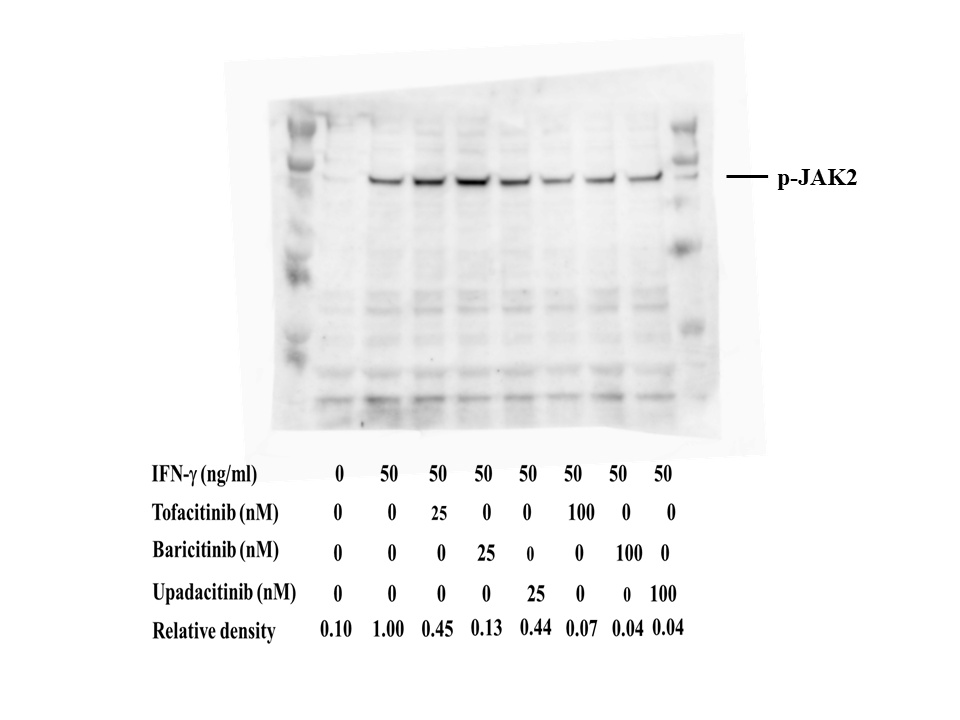

Supplement: Supplementary file 6 — Additional file 6: p-JAK2 full blot. Neutrophils were pretreated with JAKi (tofacitinib, baricitinib, upadacitinib) at the indicated concentrations for 1 h and then stimulated with IFN-γ (50 ng/ml) for 20 min. Phosphorylation of JAK2 was determined by Western blotting using phospho-specific antibodies against JAK2. [file 13104_2021_5860_MOESM6_ESM.tif]
